# Both coiling and clipping induce the time-dependent release of endogenous neuropeptide Y into serum

**DOI:** 10.3389/fneur.2023.1325950

**Published:** 2024-02-14

**Authors:** Elisabeth Bründl, Martin Proescholdt, Petra Schödel, Katharina Rosengarth, Eva-Maria Störr, Sylvia Bele, Martin Kieninger, Manuela Malsy, Nils Ole Schmidt, Karl-Michael Schebesch

**Affiliations:** ^1^Department of Neurosurgery, University Medical Center Regensburg, Regensburg, Germany; ^2^Department of Orthopedics, Trauma and Hand Surgery, Section Neurosurgery, Medical Center St. Elisabeth, Straubing, Germany; ^3^Department of Anesthesiology, University Medical Center Regensburg, Regensburg, Germany; ^4^Department of Neurosurgery, Paracelsus Medical Private University, Nuremberg, Germany

**Keywords:** biomarker, cerebrovascular manipulation, clip, coil, cognition, neuropeptide Y (NPY), outcome, unruptured intracranial aneurysms (UIA)

## Abstract

**Background:**

The vaso- and psychoactive endogenous Neuropeptide Y (NPY) has repeatedly been shown to be excessively released after subarachnoid hemorrhage and in numerous psychiatric disorders. NPY is stored in sympathetic perivascular nerve fibers around the major cerebral arteries. This prospective study was designed to analyze the impact of microsurgical and endovascular manipulation of the cerebral vasculature versus cranio- and durotomy alone on the serum levels of NPY.

**Methods:**

58 patients (drop-out n = 3; m:*f* = 26:29; mean age 52.0 ± 14.1 years) were prospectively enrolled. The vascular group underwent repair for unruptured intracranial aneurysms (UIA) of the anterior circulation [endovascular aneurysm occlusion (EV) n = 13; microsurgical clipping (MS) n = 17]; in the non-vascular group, 14 patients received microsurgical resection of a small-sized convexity meningioma (CM), and 11 patients with surgically treated degenerative lumbar spine disease (LD) served as control. Plasma was drawn (1) before treatment (t_0_), (2) periprocedurally (t_1_), (3) 6 h postprocedurally (t_2_), (4) 72 h postprocedurally (t_3_), and (5) at the 6-week follow-up (FU; t_4_) to determine the NPY levels via competitive enzyme immunoassay in duplicate serum samples. We statistically evaluated differences between groups by calculating one-way ANOVA and for changes along the time points using repeated measure ANOVA.

**Results:**

Except for time point t_0_, the serum concentrations of NPY ranged significantly higher in the vascular than in the non-vascular group (*p* < 0.001), with a slight decrease in both vascular subgroups 6 h postprocedurally, followed by a gradual increase above baseline levels until FU. At t_3_, the EV subgroup showed significantly higher NPY levels (mean ± standard deviation) than the MS subgroup (0.569 ± 0.198 ng/mL vs. 0.415 ± 0.192 ng/mL, *p* = 0.0217). The highest NPY concentrations were measured in the EV subgroup at t_1_, t_3_, and t_4_, reaching a climax at FU (0.551 ± 0.304 ng/mL).

**Conclusion:**

Our study reveals a first insight into the short-term dynamics of the serum levels of endogenous NPY in neurosurgical and endovascular procedures, respectively: Direct manipulation within but also next to the major cerebral arteries induces an excessive release of NPY into the serum. Our findings raise the interesting question of the potential capacity of NPY in modulating the psycho-behavioral outcome of neurovascular patients.

## 1 Introduction

With increasing awareness over the past few decades, vascular neurosurgery is evaluated with regard to both the patient’s functional disability and their cognitive and neuropsychological outcome (1–3). Cognitive impairment and psycho-behavioral maladaptation after cerebrovascular treatment of ruptured and unruptured intracranial aneurysms (UIA) imply a decreased quality of life and are common but still underdiagnosed (3, 4). Levels of stress accompanied by a cascade of related neuropeptidergic and endocrine responses may modulate short- and long-term psychological and physiological functioning. Peptidergic neurotransmitters have been identified as influential neuromodulators of stress-related emotionality (5).

Among the biochemical mechanisms and potential biomarkers identified, the potent vaso- and psychoactive neuropeptide Y (NPY) (6) seems to advance to a rather appealing target for further research. NPY is the most abundant and widely distributed neuropeptide in the human brain (7), significantly impacting brain activity. In the central nervous system (CNS), NPY is considerably involved in numerous behavioral and physiological processes associated with stress and stress resilience, energy homeostasis, cognition, various psychiatric disorders, pain, and the control of food intake (8, 9). A growing body of evidence attests NPY has an eminent role as a protective endogenous mediator of stress resilience, promoting a homeostatic balance to stressors and environmental changes (10–12). NPY provides a tremendous spectrum of intrinsic effects. Acting as a highly potent and prolonged endogenous vasoconstrictor, NPY physiologically restores the cerebral vascular tone (7, 13, 14) and, accordingly, the cerebral blood flow (CBF) (14). The onset of migraine was attributed to a dysregulation of its antagonistic interaction with vasodilatative neuropeptides (15). Additionally, in basic and clinical experimental research on subarachnoid hemorrhage (SAH) in animal models and in humans, NPY has repeatedly shown to be excessively released into the cerebrospinal fluid (CSF) and into serum (16–22). Compared to other types of intracranial hemorrhage, we observed an increased release of NPY into CSF to be specific for SAH (21). In this context, NPY was attested a major pathophysiological role in SAH-related cerebral vasospasm (CV) (13, 18, 20, 23–25) with excessively increased NPY levels in arterial CV and cerebral ischemia (16, 17). Beyond, upregulated NPY levels after spontaneous (i.e., non-traumatic) SAH were postulated to be associated with poorer health-related quality of life (hrQoL) domains (22). However, a deficient upregulation of NPY concentrations during stress exposure was proven equally detrimental (11, 12, 26, 27).

NPY is stored in sympathetic perivascular nerve fibers around the major cerebral arteries of the anterior arterial cerebral circulation (7, 28), that release and induce the reuptake of neuropeptides ‘on demand’ (23), and in free nerve endings in the *dura mater* (29). We hypothesize that, in patients undergoing extra- and intraluminal repair for an unruptured intracranial aneurysm (UIA), the type of treatment maneuver next to the NPY-containing perivascular nerve fibers might considerably affect NPY dysregulation. To the best of our knowledge, no study has previously investigated the potential impact and extent of the cerebrovascular manipulation on endogenous serum NPY levels over time in elective neurosurgical patients. We prospectively evaluated the treatment-specific differences in the secretion of endogenous NPY into serum during the acute stage after microsurgical and endovascular manipulation of the cerebral arterial vasculature, suggesting an excessive NPY release.

## 2 Patients and methods

The study protocol, the prospective liquid biobanking, as well as the clinical database were approved by the local institutional Ethics Committee (14–101-0010). The study complies with the Declaration of Helsinki.

### 2.1 Patient population

A part of our cohort has been reported previously (30). In this single-center trial at our University Medical Center, we prospectively enrolled patients undergoing either cerebrovascular manipulation (vascular group) during repair for UIA of the anterior circulation [endovascular aneurysm occlusion (EV subgroup) and microsurgical clipping (MS subgroup), respectively], or, a non-vascular neurosurgical procedure (non-vascular group). To control for craniotomy and durotomy alone, one non-vascular subgroup received microsurgical resection of a small-sized convexity meningioma (CM subgroup), while another subgroup of patients with surgery on degenerative lumbar spine disease (LD subgroup), i.e., lumbar disk herniation or lumbar spinal canal stenosis, served as control to eliminate side effects of general anesthesia or of the surgical procedure itself.

**Figure 1:**
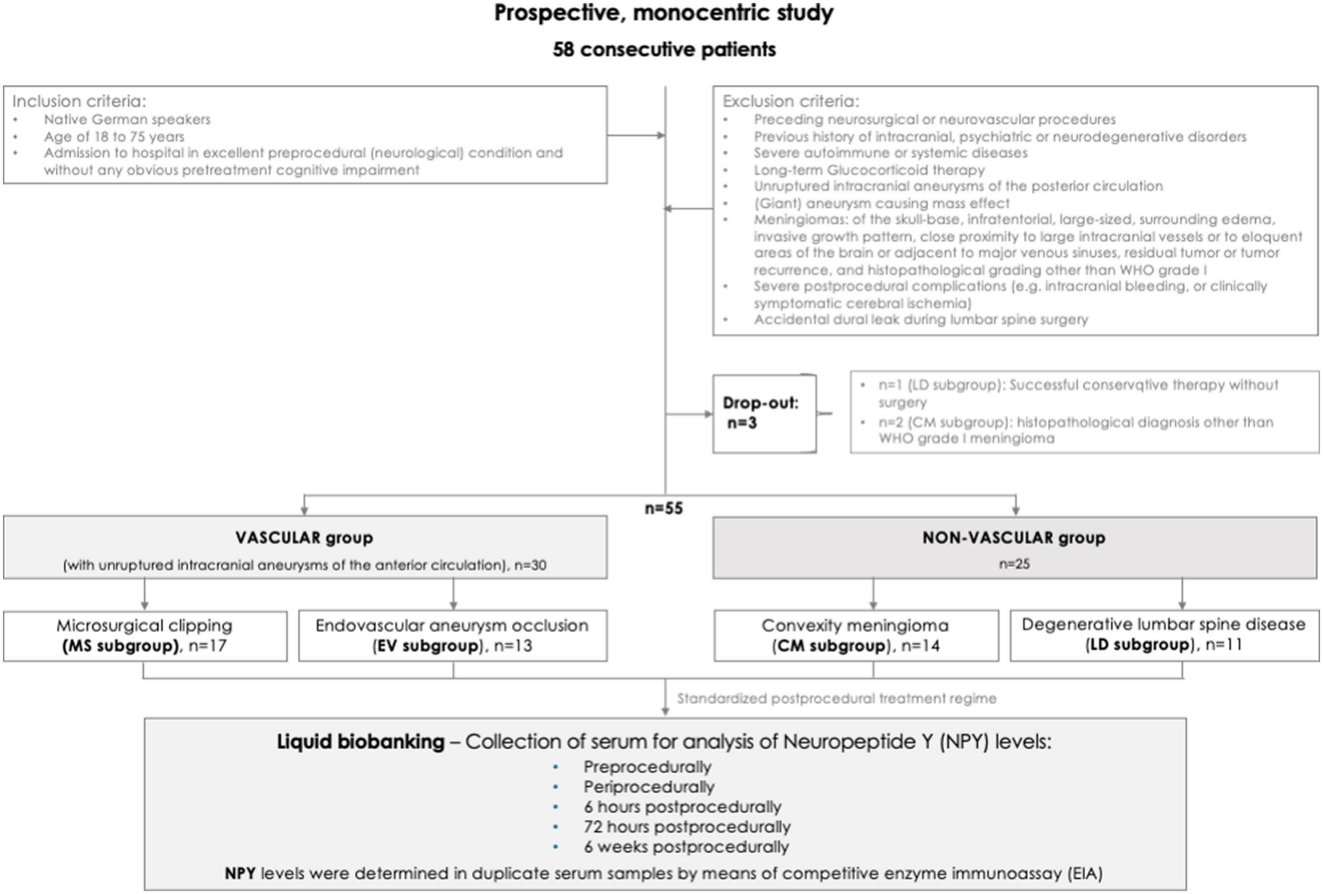
Flowchart. Study design, selection criteria, and reasons for exclusion of potentially eligible cases.

*Study selection criteria* (Figure 1). After obtaining written informed consent, we selectively included native German speakers, aged 18 to 75 years, who were admitted to hospital for endovascular or microsurgical obliteration of an anterior circulation UIA, for microsurgical resection of a convexity meningioma, or for lumbar spine surgery. In the CM subgroup, we exclusively considered patients with small-sized convexity meningioma of World Health Organization (WHO) grade I, which were located superficially in a non-eloquent brain region. A macroscopically total resection had to be confirmed (Simpson grade I-II). At admission, each patient presented in excellent preprocedural (neurological) condition and without any obvious pretreatment cognitive impairment.

Exclusion criteria were any serious postprocedural complications leading to a persistent neurological deterioration, such as intracranial bleeding or clinically symptomatic cerebral ischemia. Surgical revision alone was not considered as an exclusion criterion. Skull-base meningiomas, infratentorial meningiomas, large-sized meningiomas ( > 6.5 cm in any diameter), relevant edema surrounding the benign tumors suggesting an invasive growth pattern (except for circumscribed intraosseous osteolytic infiltration), meningiomas with close proximity to large intracranial vessels or to eloquent areas of the brain or adjacent to major venous sinuses, residual tumor according to Simpson grade III-V or short-term tumor recurrence, and meningiomas with a histopathological grading other than WHO grade I were also excluded. Further criteria for exclusion were aneurysms in the posterior circulation, and the presence of symptomatic (giant) aneurysms causing mass effect. Additionally, all patients with a previous history of intracranial disorders, preceding neurovascular or neurosurgical procedures, a previous history of psychiatric or neurodegenerative diseases, severe autoimmune or systemic diseases such as rheumatic illnesses of the musculoskeletal system, and with long-term glucocorticoid substitution were excluded from analysis.

The clinical database (see Table 1) comprised all demographic and neurological variables including neurological examinations at hospital admission, at discharge and at the 6-week follow-up (FU), comorbidities, comprehensive pharmacological screening at discharge, non−/invasive procedures, and treatment-associated complications. Outcome grading was registered before discharge and at the 6-week FU using the Glasgow Outcome Scale (GOS) (31) and the modified Ranking Scale (mRS) (32), and, for the CM patients, additionally by means of the Karnofsky performance status scale (33) and the Index of the Eastern Cooperative Oncology Group (ECOG) (34). The presenting symptoms are depicted in Table 2. Histopathological and neuroradiological details including the site and size of the CM and UIA, respectively, and the number of UIA detected can be gleaned from Table 3. The CM subgroup received a magnetic resonance imaging (MRI) pre- and (not later than 3 months) postoperatively. The vascular group underwent conventional diagnostic digital subtraction angiography (DSA) before treatment and at the FU (MS subgroup: before discharge, EV subgroup: 6 months after treatment). Additionally, within 24 h postprocedurally, a cerebral computed tomography (CT) scan was obtained.

**Table 1:** Demographical, clinical, and intra- and postprocedural patient characteristics.

Clinical features and patient characteristics	Study population			
	MS	EV	CM	LD
Number of patients [*n*]	17	13	14	11
Male to female ratio	10: 7	6: 7	3: 11	7: 4
Age [years], mean ± SD	58.1 ± 7.8	50.0 ± 13.1	53.4 ± 16.6	43.0 ± 16.0
Number of [years] of education, mean ± SD	9.2 ± 1.5	9.7 ± 1.7	9.8 ± 1.8	9.0 ± 0.4
*Levels of serum neuropeptide Y (NPY)*				
Preprocedurally [ng/ml], mean ± SD	0.378 ± 0.082	0.343 ± 0.079	0.320 ± 0.148	0.318 ± 0.095
Periprocedurally [ng/ml], mean ± SD	0.450 ± 0.264	0.540 ± 0.177	0.239 ± 0.170	0.368 ± 0.099
6 h postprocedurally [ng/ml], mean ± SD	0.379 ± 0.215	0.405 ± 0.187	0.252 ± 0.127	0.298 ± 0.111
72 h postprocedurally [ng/ml], mean ± SD	0.365 ± 0.192	0.507 ± 0.198	0.315 ± 0.215	0.311 ± 0.088
6 weeks postprocedurally [ng/ml], mean ± SD	0.518 ± 0.201	0.551 ± 0.304	0.363 ± 0.250	0.330 ± 0.137
*Handedness*				
Right (retrained)	14 (1)	10 (1)	11 (1)	10 (1)
Left (retrained)	0	1	1 (1^a^)	0
No information available	3	2	2	1
*Comorbidities*				
Arterial hypertension	11	5	5	1
Cardiac disorders	3	1	4	1
Vascular angiopathy, peripheral arterial disease	4	2	0	1
Venous thromboembolism	1	0	1	0
Abdominal aortic aneurysm	0	1	0	1
Diabetes mellitus	1	0	0	1
Adiposity	0	1	1	3
Nicotine abuse	1	0	0	0
Small convexity meningioma	1	0	1	0
Thyroid dysfunction	4	0	1	0
Asthma	0	0	0	1
Migraine, neuralgia, joint disease	3	2	4	1
*Procedure variables*				
Duration from initial diagnosis to procedure [months], mean ± SD (range)	5.7 ± 6.8 (1–28)	1.6 ± 1.1 (0–3)	18.3 ± 48.2 (0–168)	–
Duration of surgery/intervention [min], mean ± SD (range)	163.5 ± 44.0 (103–235)	122.1 ± 42.8 (42–173)	188.7 ± 98.0 (90–421)	136.2 ± 56.1 (69–250)
Temporary parent artery occlusion during UIA repair [min], mean ± SD (range)	2.4 ± 4.1 (0–15.7)	–	–	–
Time with mechanical ventilatory support [hours], mean ± SD (range)	8.3 ± 4.0(3.2–19.4)	2.8 ± 1.1(1.3–4.5)	6.2 ± 2.5(3.1–12.2)	2.9 ± 0.7(2.2–4.2)
*Complications requiring surgical revision**^b^*	1	0	4	0
*Patients with new neurological deficit [n]*				
Postoperatively	5	2	4	0
At discharge	3	1	3	0
At FU	3	1	0	0
*Treatment-associated lesions on postprocedural CT scan [n]*	5^c^	0	1^d^	–
*UIA occlusion according to postprocedural DSA*				
Complete	15	8	–	–
Incomplete (minimal rest perfusion)	3	3	–	–
Not available	1	3	–	–
*Outcome grading at discharge/at FU [n]*				
*GOS/mRS:*				
5/0	12 / 12	9 / 7	12 / 14	10 / 11
5/1	4 / 4	3 / 5	1 / 0	1 / 0
5/2	0 / 0	1 / 1	0 / 0	0 / 0
4/2	0 / 0	0 / 0	1 / 0	0 / 0
3/4	1 / 1	0 / 0	0 / 0	0 / 0
*Karnofsky Index/ECOG:*				
100/0	–	–	12 / 14	–
90/0	–	–	2 / 0	–
				
*Medication at discharge*				
Anticonvulsive drugs	2	0	6	0
Benzodiazepines / Neuroleptics	0 / 0	0 / 0	0 / 0	0 / 0
Opioid	2	2	0	3
Systemic short-term glucocorticoid therapy	1	2	3	1
Thyroid medication	5	0	6	2
Antihypertensive drugs	12	6	7	2
Antiplatelet agents (periprocedurally paused)	2 (7)	5 (0)	0 (4)	0 (2)
Nicotine patches	0	0	0	0
No medication	0	2	0	0

^a^The patient was retrained from the right to the left hand after injury of the right hand.

^b^Postoperative complications in the CM group encompassed cerebral bleeding (*n* = 1), *n* = 2 wound infections with subgaleal and epidural empyema, requiring craniectomy and cranioplasty (*n* = 3), and resection of a hypertrophic scar (*n* = 1). One MS patient underwent revision of a postoperative cranial cerebrospinal fluid fistula.

Postoperative CT scans showed (1) ^c^ a small ischemia of the caudate nucleus due to occlusion of the recurrent artery of Heubner in 5 MS patients, each, and, (2) ^d^ a circumscribed cerebral bleeding within the resection cavity in 1 CM patient 24 h postoperatively.

CM: convexity meningioma group; CT scan: computerized axial tomography scan; ECOG: Index of the Eastern Cooperative Oncology Group; EV: endovascular treatment group; FU: 6 weeks-follow-up; GOS: Glasgow Outcome Scale; LD: degenerative lumbar spine disease group (control); min: minutes; MRI: magnetic resonance imaging; mRS: modified Ranking Scale; MS: microsurgical clipping group; NPY: neuropeptide Y; SD: standard deviation; UIA: unruptured intracranial aneurysm.

**Table 2:** Presenting symptoms leading to the diagnosis of convexity meningioma (CM; *n* = 14) and to the diagnosis of unruptured intracranial aneurysms (UIA; *n* = 30) in the study population.

Clinical symptoms	[*n*]	
	CM	UIA
*Neurological deficits*		
Headache	4	5
Epileptic seizure, absence seizure, syncope	5	5
Aphasia	1	1
Transient paresthesia	2	1
Transient paresis	0	2
Vertigo	3	4
Cranial nerve (CN) deficits (CN III, V)	0	2
Transient diplopia, transient visual defect	1	3
Obliviousness, confusion	1	4
Weak concentration	0	1
		
*Incidental findings due to other diseases*		
Arterial hypertension, coronary heart disease, carotid stenosis	0	4
Transient ischemic attack	0	2
Sinusitis	1	0
Trauma	0	1
Tumor follow-up: hypopharynx carcinoma, squamous cell cancer	0	2
Vestibular neuritis, pulsatile tinnitus, hypoacusis	3	1
		
*Family history of subarachnoid hemorrhage*	0	1

CM: convexity meningioma; CN: cranial nerve; UIA: unruptured intracranial aneurysms.

**Table 3:** Aneurysm characteristics and convexity meningioma characteristics.

Aneurysm characteristics	MS (*n* = 17)	EV (*n* = 13)
**Aneurysm location**		
*Single aneurysms [n]*		
ICA, PCoA	3	6
ACA, ACoA, pericallosal artery	4	6
MCA	10	1
*Multiple aneurysms [n]*		
ACoA + MCA	2	0
ICA* + MCA	1	0
MCA (bilateral)	1	0
PCoA + ACoA	0	1
**Total number of aneurysms [n]**		
ICA, PCoA	4	7
ACA, ACoA, pericallosal artery	5	6
MCA	12	1
	21	14
**Side of aneurysms [*n*]**		
Left	7	5
Right	10	8
		
**Aneurysm size [mm], mean (± SD)**		
Maximum craniocaudal diameter	4.7 (± 2.1)	5.3 (± 2.9)
Maximum diameter in width	4.6 (± 2.2)	4.0 (± 1.5)
Convexity meningioma characteristics	CM [*n*]	
**Meningioma location**		
Frontal	7	
Fronto-temporal	1	
Centro-parietal	1	
Fronto-temporo-parietal	1	
Parieto-occipital	3	
**Side of convexity meningioma**		
Left	9	
Right	6	
**Tumor volume [cm** ^**3**^ **], mean (± SD)**	14.5 (± 12.2)	
**Meningioma size**		
Maximum axial diameter [cm], mean (± SD)	3.3 (± 1.5)	
Maximum craniocaudal diameter [cm], mean (± SD)	2.7 (± 0.7)	
**Gross total resection** (according to postoperative MRI)	14	
	
**Simpson grade**		
°I	12	
°II	2	
	
**Histological grading**		
**WHO grade I**	14	
*Histological subtype:*		
Transitional	5	
Angiomatous	1	
Microcystic-angiomatous	1	
Fibrous	3	
Meningothelial	3	
Secretory	1	
**Ki-67 proliferation index/MIB1 [%]**		
3%	1	
4%	4	
5%	9	

ACA: anterior cerebral artery; ACoA: anterior communicating artery; CM: convexitiy meningioma; EV: endovascular treatment group; ICA: internal carotid artery; ICA*: ICA-aneurysm occlusion was electively conducted in a second surgical intervention after clipping of the MCA aneurysm and not within the 6 weeks of follow-up; Ki-67 proliferation index/MIB1: typical immunohistochemical marker for cell proliferation. MCA: middle cerebral artery; MRI: magnetic resonance imaging; MS: microsurgical clipping group; PCoA: posterior communicating artery; SD: standard deviation; WHO: World Health Organization.

### 2.2 Liquid biobanking and laboratory procedures

For comparative analysis of the levels of NPY, serum was drawn at five different points in time: Blood samples were taken (1) the day before surgery/intervention (t_0_), (2) periprocedurally (t_1_), (3) 6 h postprocedurally (t_2_), (4) 72 h postprocedurally (t_3_), and (5) 6 weeks postprocedurally (t_4_; at the FU). At the time points t_1_ and t_2_, serum was drawn directly from the arterial line, at the time points t_0_, t_3_, and t_4_, and in the LD subgroup at all time points, blood was collected by venipuncture once daily, preferentially in the morning. Glucocorticoid treatment was avoided whenever possible. In cases of postprocedural nausea and vomiting or in patients with moderate postoperative cerebral edema, glucocorticoid administration was restricted to a low dose of 4 mg dexamethasone (with a maximum daily dose of 12 mg not exceeding 7 days). Immediately subsequent to sampling, the probes were centrifuged at 1200 rounds per minute for 10 min, and the supernatants were aliquoted and stored at −80°C until further use. The samples were thawed, aliquoted (1 mL) with columns [STRATA C18-E (55 μm, 70 A) 200 mg/3 mL-columns, Phoenix Pharmaceuticals Inc., Burlingame, USA], purified, evaporated on a vacuum concentrator (Christ RVC 2–25 CD plus; Osterode am Harz, Germany), and dissolved in 250 μL of assay-buffer resulting in a fourfold concentration. NPY levels were measured in duplicate purified serum samples using a competitive enzyme immunoassay (EIA; Phoenix Pharmaceuticals Inc., Burlingame, CA, USA). The cerebral exposure to the endogenously released NPY into serum over time was measured as mean ± standard deviation (SD) and expressed as [ng/ml].

### 2.3 Therapeutic procedures

In each CM patient and in each patient of the vascular group, the individual treatment modality (watch and wait vs. CM resection and EV vs. MS, respectively) was evaluated by multi-disciplinary consensus at our neurooncological tumor board, and, by neurosurgeons and neuroradiologists, at our neurovascular board, respectively.

Microsurgical and endovascular procedures in this study were provided by equally experienced senior physicians. Standard microsurgical techniques were used for UIA repair and for convexity meningioma resection, our respective standardized microsurgical and endovascular procedure protocols have been described elsewhere (30). UIA clipping was performed by three specialized vascular neurosurgeons (head physician and senior physicians) with over ten to 30 years of experience in aneurysm surgery. In our institutional neurovascular center, 80 patients per year receive endovascular and microsurgical aneurysm occlusion, respectively, with an annual volume of 20 patients undergoing clipping for both ruptured and unruptured aneurysms. Endovascular devices for UIA repair encompassed coiling and, in the case of wide neck and large or fusiform UIA, a stent-assisted system, web device, or flow diverter implantation. For convexity meningioma resection, conventional craniotomy was followed by sharp opening of the dura and tumor resection with excision of the tumorous dural insertion zone. Preferentially, the dura was replaced with periosteum, or, if not available, with artificial dura like Duragen® or Neuropatch®. Postoperatively, the CM subgroup and the vascular group underwent standardized intensive care treatment over night (35). In the LD subgroup, conventional interlaminar fenestration with decompression or sequester−/discectomy was performed for lumbar spinal canal stenosis and for lumbar disk herniation, respectively. The corresponding procedure variables are summarized in Table 1.

### 2.4 Statistical analysis

Continuous data are presented as mean ± SD and range (minimum to maximum), and categorical data as frequency counts. Correlations between mean NPY concentrations and clinical variables were calculated using t-test for two group comparison (gender and lesion on right/left side), an analysis of variance (Bartlett’s test for equal variances) for multiple group comparison (location of aneurysms). Correation between NPY values and clinical parameters showing continous variables (age, surgery time, anesthesia time) were conducted by calculating a Pearson correlation analysis.

Intergroup variances were evaluated by calculating one-way analysis (ANOVA) with Bonferroni correction for multiple testing. Changes of the mean NPY concentrations over time within the vascular and the non-vascular group and within each subgroup, respectively, were analyzed by calculating repeated measure ANOVA. Differences between subgroups were analyzed by using an analysis of variance (ANOVA) followed by Bonferroni post-hoc pairwise comparisons and adjustment by the Bonferroni correction for multiple tests. Statistical analysis was conducted according to Stata procedures (Stata Version 16.1; Stata Corp. College Station, TX, United States).

A value of *p* <0.05 was considered as statistically significant.

## 3 Results

### 3.1 Demographics and descriptive statistics

Secondarily, three of the initially acquired 58 patients were excluded from analysis, because they did not comply with the inclusion criteria: One LD patient was discharged without surgery after successful conservative therapy for a degenerative lumbar disk protrusion, and, in the CM subgroup, one patient each received the diagnosis of a histopathological WHO grade II meningioma and, the unexpected histopathological diagnosis of a cavernoma, respectively. Pursuing our strict selection criteria, a total of 55 patients (male: female = 26: 29; mean age 52.0 +/− 14.1 years; range 24 to 74 years; scholar education 9.3 +/− 1.3 years) were included in our study. The vascular group encompassed 30 patients with anterior circulation UIA (EV subgroup *n* = 13; MS subgroup *n* = 17). UIA treatment consisted either of microsurgery via pterional craniotomy and clipping (MS subgroup: clip *n* = 12; clip in combination with wrap *n* = 3; wrap *n* = 2) or intervention (EV subgroup: coil *n* = 8; balloon-assisted coil *n* = 1; flow diverter *n* = 1; web device *n* = 2). No aneurysm ruptured periprocedurally. 14 patients in the non-vascular CM subgroup received microsurgical resection of a small-sized, supratentorial convexity meningioma, and another 11 patients with lumbar spinal canal stenosis (*n* = 2) and lumbar disk herniation (*n* = 9), respectively, had surgery on a degenerative lumbar spine disease (LD subgroup for control). In the CM subgroup, 5 tumors showed an infiltrative intraosseous growth pattern, two of whom required a cranioplasty in the context of tumor resection. Pronounced cortical tumor adherence was reported in 4 CM patients.

Concerning periprocedural complications, n = 3 EV patients presented with transient, hemodynamically non-relevant thromboembolic events requiring lysis therapy, *n* = 1 EV patient, each, had a temporary cerebral vasospasm and a dissection of the internal carotid artery (ICA) requiring therapeutic anticoagulation, respectively. Due to a vascularized meningioma, one CM patient required a procedure-related blood transfusion, and one CM patient with moderate perioperative brain edema required osmotic therapy.

Postprocedural CT scans revealed treatment-associated lesions in 6 patients (small ischemia of the caudate nucleus due to occlusion of the recurrent artery of Heubner in 5 MS patients, each, and a circumscribed cerebral bleeding within the resection cavity in 1 CM patient 24 h postoperatively). No patient developed serious cerebral ischemia. Postprocedurally, 11 patients presented with a new neurological deficit (MS *n* = 5, EV *n* = 2, CM *n* = 4): In the MS subgroup, 3 patients presented with a new hemi−/paresis (with immediate recurrence in n = 2 patients), 1 patient had hemifacial paresthesia, and 1 patient reported subjective short-term memory impairment. In the EV subgroup, 1 patient had a transient speech disorder (recurrent within hours), and 1 patient reported a subjective long-term memory disorder. In the CM subgroup, 1 patient had a recurrent foot extensor paresis, 2 patients presented with a completely recurrent hemiparesis and dysarthria, which dissipated within 48 h. Postoperative complications, requiring revision (MS *n* = 1, CM *n* = 4), encompassed cerebral bleeding (CM *n* = 1) and wound infections with subgaleal and epidural empyema (CM n = 2: no microbial proof of germs and detection of *Staphylococcus epidermidis* in one patient each), respectively, each requiring craniectomy and cranioplasty (*n* = 3), and resection of a hypertrophic scar (CM *n* = 1). One MS patient with a postoperative CSF fistula and CSF infection (with the detection of *Staphylococcus epidermidis*) required a lumbar drainage. In all patients, functional outcome was stable or even improved over the 6-week FU (MS *n* = 3, EV *n* = 1, CM and LD *n* = 0, each). No CSF shunt was required, and no late rebleeding or mortalities had occurred until FU. No patient got lost to the 6-week FU.

Table 1 shows the detailed baseline data, Table 2 the presenting symptoms, and Table 3 the aneurysm and meningioma characteristics, respectively. It is worth mentioning that, surprisingly, the majority of our cohort underwent clipping for anterior circulation UIA, which is not representative as, in principle, our neurovascular center follows the general trend toward a ‘coiling first policy’ (36).

Statistical intergroup comparisons yielded a higher number of middle cerebral artery (MCA) aneurysms in the MS subgroup and a longer mean time spent on mechanical ventilatory support in the MS subgroup compared to the EV subgroup (*p* < 0.001), in the CM subgroup than in the EV subgroup (*p* = 0.012), in the MS subgroup than in the LD subgroup (*p* < 0.001), and in the CM subgroup than in the LD subgroup (*p* = 0.024). The other clinical variables did not differ significantly between the groups. There was no significant difference in the mean NPY concentrations between gender and side of the lesion over the entire observation period. Also, no significant correlation was found between NPY concentrations and age, duration of intervention, anesthesia time, or outcome parameters such as GOS and mRS, neither at discharge nor at FU.

### 3.2 NPY dynamics in serum in the short-term after manipulation on the central and peripheral nervous system

#### 3.2.1 Intergroup analysis

Preprocedurally (at t_0_), the mean NPY concentrations did not differ significantly between the sub−/groups (mean total NPY at t_0_: vascular 0.373 ± 0.080 ng/mL vs. non-vascular 0.328 ± 0.126 ng/mL). At all other assessment intervals (time points t_1_-t_4_), the mean serum concentrations of NPY ranged significantly higher in the vascular than in the non-vascular group (*p* < 0.001; t_1_: *p* = 0.0001; t_2_: *p* = 0.0003; t_3_: *p* = 0.002; t_4_: *p* = 0.0049). Periprocedurally, an upregulation of the NPY concentrations was detected in both vascular groups (EV: 0.540 ± 0.177 ng/mL; MS: 0.521 ± 0.264 ng/mL; non-sig. *p* = 0.292) and, moderately in the LD subgroup, whereas in the CM subgroup, the NPY levels gently declined intraoperatively. In both vascular subgroups, the periprocedural NPY peak was followed by a slight decrease in NPY secretion 6 h postprocedurally. At 72 h postprocedurally, the EV subgroup showed significantly higher NPY levels than the MS subgroup (0.569 ± 0.198 ng/mL vs. 0.365 ± 0.415 ng/mL, *p* = 0.021). From 6 h postprocedurally (t_2_) onwards, the EV subgroup, more than both non-vascular groups, demonstrated a gradual increase above baseline levels until FU.

#### 3.2.2 Intragroup analysis

The highest mean NPY concentrations were measured in the EV subgroup at the time points t_1_, t_3_, and t_4_, reaching a climax at FU (mean NPY at t_4_: 0.551 ± 0.304 ng/mL). Further intragroup analyses revealed the highest mean NPY levels in the CM subgroup at FU (t_4_), in the MS subgroup, and in the LD subgroup intraoperatively (at t_1_), respectively. Interestingly, in the vascular group, we observed a significant increase in NPY concentrations during the procedure both in the MS as well as in the EV group (*p* = 0.030 and 0.016, respectively). In contrast, in both the LD and the CM subgroup, no such dynamic was observed (*p* = 0.412 and 0.390, respectively). Comparing t_1_-t_4_ against t_0_ as control (repeated measure ANOVA, *p* < 0.05), significantly increased NPY concentrations were measured in the MS subgroup at t_1_, leveling down to values not significantly different from those preprocedurally (at t_0_). In contrast, in the EV subgroup, the NPY levels also increased significantly at t_1_, however, they plateaued on a significantly elevated level throughout the entire observation period.

**Figure 2:**
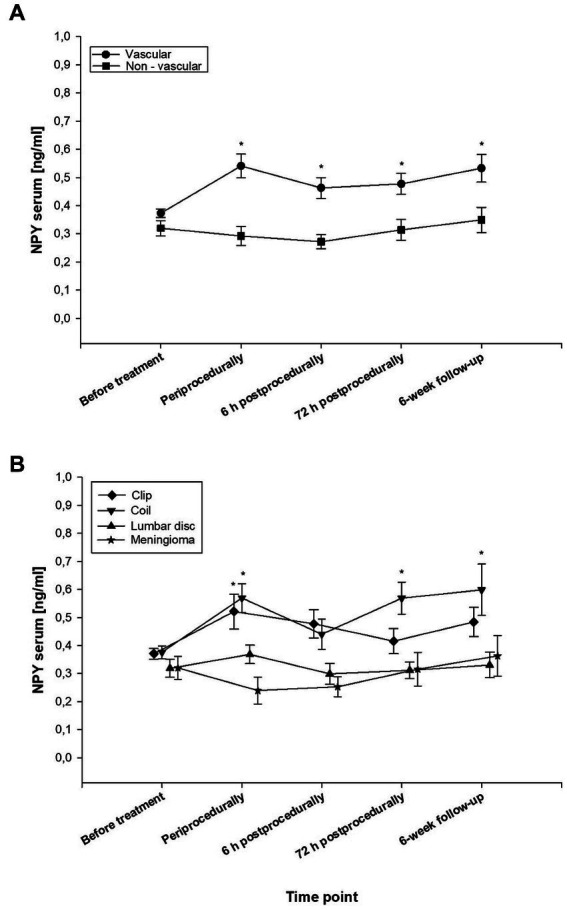
Temporal dynamics of endogenous neuropeptide Y (NPY) levels in serum, plotted against the different types of manipulation on the central and peripheral nervous system. The line graph with error indicators displays the treatment-dependent dynamics of mean values of NPY in serum over time (t_0_: before treatment, t_1_: periprocedurally, t_2_: 6 h postprocedurally, t_3_: 72 h postprocedurally, and t_4_: at the 6-week follow-up (FU) in **(A)** the vascular versus the non-vascular group, and in **(B)** all four subgroups: **(A):** The black line chart with circles represents the vascular group, the squares the non-vascular group. The vascular group showed significantly higher NPY values compared to the non-vascular group in all time points except t_0_ (*p* < 0.05). **(B):** Intragroup comparison of endovascular aneurysm occlusion (EV) subgroup (triangle down), microsurgical clipping (MS) subgroup (diamond), convexity meningioma (CM) subgroup (stars), and degenerative lumbar spine disease (LD) subgroup (triangle up). The asterisks indicate *p*-values smaller than 0.05, resulting from the repeated measure ANOVA, comparing t_1_-t_4_ against t_0_ as control. The MS subgroup displays significantly increased values on t_1_, but levels down to values not significantly different compared to t_0_. In contrast, the EV subgroup also increases significantly on t_1_, but stays higher throughout the entire observation period. Neither the CM nor the LD subgroup showed any significant change compared to t_0_. Each symbol displays the mean level of serum NPY in [ng/ml] for each (sub-)group, indicating significantly higher mean NPY concentrations in the vascular group from t_1_ onward. At t_3_, the EV subgroup showed significantly higher NPY levels than the MS subgroup (*p* = 0.021). Statistical significance: *p < 0.05.

The treatment-dependent NPY concentrations in serum over time are displayed in Figure 2. The respective continuous data are summarized in Table 1.

## 4 Discussion

In the present study, we focused on the following three paramount questions: (1) Does cerebrovascular manipulation induce the release of endogenous NPY into serum?, (2) Are there treatment-specific differences in the NPY secretion during the acute stage after microsurgical and endovascular manipulation of the cerebral vasculature compared to non-vascular neurosurgical procedures, and (3) Do these concentrations vary over time?

### 4.1 Cerebrovascular manipulation imparts a strong stimulus for NPY release into serum

Our results corroborate the hypothesis that UIA treatment notably affects NPY dysregulation. As an explanatory scientific approach, it could be reasoned, that the NPY upregulation in serum might be induced by the type of treatment maneuver next to the NPY-containing perivascular nerve fibers. Preprocedurally, the NPY concentrations did not differ significantly between the subgroups. Non-vascular microsurgery for convexity meningioma and lumbar degenerative spine disease did not significantly alter the amount of NPY in serum during the first 42 postoperative days. In contrast, during UIA treatment, NPY was released excessively into serum, with NPY values still elevated 6 weeks after the respective neurovascular procedure.

#### 4.1.1 Pathophysiological considerations

Since the early 1990ies, several authors scrutinized the (pathophysiological) interrelations of SAH with the potent vasoconstrictor NPY, and its impact on CV, consecutive cerebral ischemia, and, due to its multiple intrinsic and psychoactive properties, on neurobehavioral outcome in experimental animal models as well as in humans [*cf.* (22) and (21) and references within]: Considering NPY to be a promising biomarker in neuroscience and, predominantly, in SAH, it was tracked in remarkable concentrations immunohistochemically in the perivascular nerve fibers of the major cerebral arteries (25) and in the supratentorial *dura mater* (24), respectively, in CSF (16–18, 21, 22, 37), and in the external jugular vein/serum (17, 19–21, 37). The question of how cerebrovascular manipulation can exert proven neuropeptidergic changes is a matter of speculation (*cf.* Discussion section 4.1.3). Experimental SAH in various animal species resulted in both acute and chronic reductions in the density of NPY-like immunoreactive perivascular fibers. Mechanistically, it has been repeatedly suggested (16, 17, 21, 24, 25) that either direct mechanical and/or chemical stimulation might cause local damage to the NPY-containing sympathetic axons at the perivascular sites (7). It might further be speculated that, pathophysiologically, as a consequence of altered NPY synthesis and metabolism, the elevated serum NPY might reflect an arrest of axonal peptidergic transport, resulting from a local damage to axons at the perivascular sites during UIA treatment, a degenerative neuronal process, a decreased NPY production in the relevant neurons, and/or a stress-mediated central mechanism. The impact of the anatomical localization of the UIA might be discussed as well.

The present study is the first to substantiate that the cerebrovascular manipulation *per se* induces NPY secretion into serum. In our previous series on NPY dysregulation in SAH patients, the possible influence of surgical intervention (e.g., insertion of an external ventricular drain, aneurysm treatment, or hematoma evacuation) could not be excluded (16, 21). Compared to our recent findings, the serum NPY levels of a historic population (21) with different types of non-traumatic intracranial hemorrhages (n = 66 patients with aneurysmal SAH and n = 13 patients with basal ganglia hemorrhage and cerebellar hemorrhage, respectively, undergoing a daily assessment of NPY concentrations in serum and CSF over a ten-day period after ictus) ranged significantly lower than our vascular group (*p* = 0.00013). Hypothetically, a prolonged perioperatively extraluminal and periinterventionally intraluminal cerebrovascular manipulation represents a more intense stimulus for an excessive NPY release from the NPY-containing perivascular nerve fibers around the major cerebral vessels than the ‘temporary’ moment of the aneurysm and vascular wall rupture (of the *Tunica intima*, *Tunica media*, and *Tunica adventitia*), respectively (at least into serum).

#### 4.1.2 The temporal course of NPY hypersecretion

Information on the temporal dynamic changes of NPY concentrations in serum and CSF is scarce and, if provided, limited to the short term (16, 18, 21, 22, 25). In the non-vascular group, the NPY dynamics did not prove to follow a distinct course: The NPY values did neither differ significantly from each other (t_0_-t_4_) nor between both the CM and the LD subgroup. After a periprocedural NPY peak, the vascular group demonstrated a moderate decrease in NPY secretion up to 6 h postprocedurally in the EV subgroup and up to 72 h postoperatively in the MS subgroup, respectively, each followed by a steady increase above baseline levels until FU. Endoluminal manipulation alongside the endothelium of the cerebral vasculature yielded the highest serum NPY concentrations, with peak levels obtained at the assessment points t_1_, t_3_, and t_4_, reaching a climax at FU. While a previous analysis (16) of CSF NPY in SAH dismissed the influence of the treatment modality (EV vs. MS) on NPY secretion, our findings in UIA treatment (without any confounders like intracranial hemorrhage, CV, or additional surgical interventions) prove the contrary. Therapeutic cerebrovascular interference results in hypersecretion of NPY into serum for at least six weeks after the respective treatment, presumably all the more so in (and 72 h after) endoluminal UIA occlusion.

#### 4.1.3 Main source for NPY biobanking

Beyond its storage in several limbic and cortical regions, in sympathetic perivascular nerve fibers around cerebral arteries, and in the spinal cord (7, 11, 28, 38), NPY was identified in the adrenal gland (39, 40), in the intrinsic cardiac neurons, and the myenteric plexus of the gut [*cf.* references in (41)]. NPY has been found to be colocalized with norepinephrine (NE) in the adrenal medulla, in peripheral noradrenergic neurons, and in various (stress regulatory) areas of the brain (11). Central and peripheral NPY has been reported to regulate the release and activity of NE (42). In the central and peripheral sympathetic nervous system, the neurotransmitter NPY is co-released with NE during sympathetic activation (43–45), exerting stress-induced, intense regional (splanchnic, coronary, and cerebral) vasoconstriction (41), regulating the peripheral vascular resistance, as well as – together with other neuropeptides – the [cerebro-(7, 13, 14)] vascular tone, and, hereby, the CBF (11, 14, 41). The potent and prolonged vasoconstrictive effect of NPY on the cerebral vasculature *in vitro* and *in vivo* even surpasses the potency of NE (46). Thus, NPY is considered the most potent endogenous vasoconstrictor (14, 47). Intracarotid or intraparenchymal administration of NPY has been demonstrated to induce a significant reduction of CBF (14). Following SAH, NPY was attributed a pivotal role in SAH-induced CV (13, 18, 20, 23–25) and cerebral ischemia (16, 17).

The best source of NPY acquisition is still to be questioned and might potentially depend on the underlying primary disease, as, for CV monitoring in SAH patients, for example, our previous research (16, 21) suggested liquid biopsies from CSF. In the above-mentioned historic cohort with intracranial hemorrhage (16, 21), the endogenous NPY levels in serum during the first ten days after ictus did neither differ significantly between the treatment groups nor from the preoperative serum NPY concentrations of a control group without any manipulation on the central or peripheral nervous system (16, 17, 21). In CSF, however, the day-by-day release of NPY was significantly increased in the SAH group compared to the intracerebral hemorrhage group and the control group, indicating that the hypersecretion of NPY into CSF, but not into serum, is specific for SAH (21).

In the present study, methodological and ethical concerns hindered us from evaluating the NPY levels in CSF, since this specific cerebrovascular cohort (without hydrocephalus) is not amenable to CSF diagnostics. Future experimental research is demanded, to identify the primary sources for NPY biobanking.

While, in patients with SAH, the supposed origin of excessive NPY release has repeatedly been postulated (16, 17, 21, 24, 25), in neurosurgical and other neurovascular patients, like patients with UIA, CM, or LD in our cohort, respectively, the potential source/s of serum NPY have not been investigated before. Sophisticated theories (16, 17, 21, 24, 25) state that focal stimuli like SAH or, as to our hypothesis, cerebrovascular manipulation, induce either an excessive release and/or a subsequent block of reuptake of NPY at the perivascular axon terminals (25) or a central neural mechanism might lead to a stress-induced sympathetic NPY activation (as it is known to follow SAH) (48). The upregulation of NPY expression, following repeated exposure to stress, might reflect an adaptive mechanism to manage stress (49). The aneurysm treatment-induced mechanical manipulation of the parent vessel itself might represent such a stressful stimulus. Confirmatory evidence for this speculation is lacking, however.

In addition to these etiological aspects, the potential interrelation between NPY concentrations, NE levels (as a parameter of sympathetic hyperreactivity), and potentially NPY-related hemodynamic changes in neurosurgical patients, and in patients with cerebrovascular malformations in particular, would be an interesting issue to be addressed in the future. Our study design did neither involve a monitoring of the NE levels in serum nor an assessment of the blood pressure profile, exceeding the standardized daily routine surveillance of all vital parameters during pre-, peri- and postprocedural intensive care and normal care treatment, respectively. Consecutively, the presented data do not suffice to provide sustainable information on the potential treatment-dependent changes in blood pressure or its notional correlation with alterations in the NPY values. However, based on the clinical database available, and on our clinical long-term experience, no striking hemodynamic intergroup differences between the MS and the EV group, for example, were obvious.

#### 4.1.4 NPY in the context of functional outcome and neurobehavioral implications

The vascular and the non-vascular group of our cohort did not vary in terms of their functional outcome (as to the GOS and mRS). In patients with aneurysmal SAH, NPY was proposed as a possible positive predictor of outcome (16, 19). In contrast, in 2018, we found upregulated NPY levels after SAH, related to poorer hrQoL (as to psychological health, depression, anxiety and nutrition disorder) (22).

The concentrations of NPY in the human brain are higher than those of any previously discovered neuropeptides with a widespread distribution in brain areas implicated in psychopathology, such as several limbic, hypothalamic, brainstem, and cortical regions (7). Tissue concentrations of NPY seem to correlate well with the density of fiber and terminal networks (38). The neurotransmitter and neurohormone NPY is intimately linked with sympathetic nerve function, and, due to its upregulation during increased sympathetic tone, it is considered a specific marker of sympathetic activity (50), as well as a key component in the intrinsic stress response systems (10). Reports on chronic stress-induced hypersecretion of NPY in the brain emphasize the implications of NPY in stress-induced psychopathology (11).

NPY is decisively involved in modulating the stress response systems like the hypothalamic–pituitary–adrenal axis system and, thereby, in the regulation of stress and anxiety, trauma-induced disorders, depression, chronic fatigue syndrome, cognition, learning and memory, information handling, neuroprotection, circadian rhythm, energy homeostasis, food intake, sexual behavior, migraine, epilepsy, schizophrenia, alcoholism, and addiction (10, 11). Physiologically, NPY tones down CNS activity: During the past two decades, several excellent reviews (12, 27, 51–54) have repeatedly substantiated the contribution of the NPY stress-integrative circuitry in attenuating the effects of stress by inhibiting the activity of the pro-stress transmitters NE and the corticotropin-releasing hormone (CRH) system (10), and, thereby, imparting anxiolysis and stress resilience (10). Severe or prolonged stress stimulates circulating serum levels of NPY and modulates NPY inactivation. Given the role of NPY in restraining noradrenergic system reactivity, under pathophysiological conditions, a dysregulation in NPY may result in enhanced NE-mediated hyperarousal (11). High levels of serum NPY may reflect the attempt to set up an endogenous neuroprotective mechanism to counteract a potential ‘degenerative’ process (55). Both preclinical and clinical data have demonstrated an upregulation of NPY to be associated with resilience in the face of extreme psychological stress (56), while, reciprocally, low NPY concentrations in CSF and serum are related to posttraumatic stress disorder, affective disorders, dementia, and a history of attempted suicide (12, 26, 27). Accordingly, establishing the relevance of NPY in neurobehavioral and cognitive outcome after neurovascular treatment is rather tempting. Considering these multifaceted interrelations, our findings raise the interesting question of whether NPY overexpression following cerebrovascular manipulation might affect the cognitive, psychobehavioral, or emotional outcome of patients with cerebrovascular malformation, which appears all the more feasible against this background. In accordance with psychobehavioral literature on NPY (12, 16, 19, 26, 27, 56), neurovascular patients with elevated postprocedural NPY concentrations might theoretically have a more favorable cognitive and neuropsychological prognosis than patients with persistently low NPY levels or with a delayed NPY increase several days after the respective treatment maneuver. To date, the behavioral profile of the action of NPY has insufficiently been characterized, though, with no data available on patients with cerebrovascular malformations.

It remains highly speculative whether, by NPY hypersecretion, the human peptidergic stress response systems endeavor to promote resilient coping strategies, attenuating stressors like cerebrovascular manipulation, or whether UIA treatment reflects such an overwhelming stimulus that NPY fails to tone down the excitatory effects of pro-stress neurotransmitters with potentially adverse psychopathological consequences.

The neuroanatomical circuitry involved in NPY transmission and modulation has to be elucidated as do its (psycho-behavioral) interactions and its temporal dynamics.

### 4.2 Methodological considerations

The limited sample size with three patients excluded may potentially have impacted the results. Selectively analyzing the endogenous NPY expression into serum might not reflect the possibly higher NPY release into CSF, as previously proven in patients with spontaneous SAH (22) and aneurysmal SAH (16, 21). However, by nature, non-hydrocephalus patients with the diagnosis of UIA or meningioma are not amenable to CSF biomarker sampling. The best source of acquiring predictive NPY values is still to be scrutinized. In addition, the present study was not designed to provide (e.g., immunohistochemical) evidence, that manipulation of the cerebral vasculature represents a direct mechanical stimulus for the NPY release from the nerve-ending terminals of the perivascular nerve fibers, surrounding the major cerebral arteries. Accordingly, the NPY concentrations in CSF, the long-term course of NPY exposure, and its influence on the CRH system, as well as on the psychopathology in neurosurgically and neuroradiologically treated patients with cerebrovascular malformations remain to be evaluated further.

The actual strength of our study, revealing the first insight into this interesting issue, lies in its standardized, controlled design with a reasonable sample size, adjustment for potential confounders, prospective data evaluation, and serial liquid biobanking, respectively.

## 5 Conclusion

Our prospective study provides the first data available, addressing the serum concentrations of endogenous NPY in neurosurgical and neurovascular patients, respectively. The release of endogenous NPY into serum was significantly increased during the first six weeks after UIA treatment. Our results impressively demonstrate the impact of direct endoluminal and extraluminal cerebrovascular manipulation of cerebral arteries on the hypersecretion of this potent vaso- and psychoactive neuropeptide. Elucidating the neuroanatomical, pathophysiological, and pathopsychological interactions of the NPY system with treatment maneuvers within or next to the cerebral vasculature in future studies will substantially advance our knowledge regarding the functional outcome and neurobehavioral recovery in patients with cerebrovascular malformations. Perspectively, as a promising novel diagnostic and treatment approach, the neuromodulator NPY and its neuropeptidergic response circuitry might provide innovative answers to the potential neurocognitive vulnerability of this specific patient population.

## Data availability statement

The original contributions presented in the study are included in the article/supplementary material, further inquiries can be directed to the corresponding author.

## Ethics statement

The study involving humans was approved by the Ethics Committee at the University of Regensburg (Study approval number: 14–101-0010). The study was conducted in accordance with the local legislation and institutional requirements. The participants provided their written informed consent to participate in this study. The study follows the principles of the Declaration of Helsinki.

## Author contributions

EB: Data curation, Investigation, Writing – original draft, Writing – review & editing, Formal analysis, Project administration, Validation, Visualization. MP: Formal analysis, Validation, Writing – review & editing. PS: Data curation, Investigation, Project administration, Writing – review & editing. KR: Project administration, Writing – review & editing. E-MS: Writing – review & editing. SB: Writing – review & editing. MK: Writing – review & editing. MM: Writing – review & editing. NS: Writing – review & editing. K-MS: Conceptualization, Methodology, Supervision, Validation, Writing – review & editing.
